# Pharmacogenetic Testing for the Pediatric Gastroenterologist: Actionable Drug–Gene Pairs to Know

**DOI:** 10.3390/ph16060889

**Published:** 2023-06-16

**Authors:** Tracy Sandritter, Rachel Chevalier, Rebecca Abt, Valentina Shakhnovich

**Affiliations:** 1 Division of Clinical Pharmacology/Medical Toxicology and Therapeutic Innovation, Children’s Mercy Hospital, 2401 Gillham Road, Kansas City, MO 64108, USA; vshakhnovich@ironwoodpharma.com; 2 Department of Pharmacy Practice, School of Pharmacy, University of Missouri-Kansas City, Kansas City, MO 64108, USA; 3 Division of Gastroenterology, Children’s Mercy Hospital, 2401 Gillham Rd., Kansas City, MO 64108, USA; rlchevalier@cmh.edu; 4 Department of Pediatrics, School of Medicine, University of Missouri-Kansas City, Kansas City, MO 64108, USA; 5 ProPharma Group, Overland Park, KS 66210, USA; rebecca.abt@propharmagroup.com

**Keywords:** pharmacogenetics, pharmacogenomics, gastroenterology, drug–gene pairs

## Abstract

Gastroenterologists represent some of the earlier adopters of precision medicine through pharmacogenetic testing by embracing upfront genotyping for thiopurine S-methyltransferase nucleotide diphosphatase (*TPMT*) before prescribing 6-mercaptopurine or azathioprine for the treatment of inflammatory bowel disease. Over the last two decades, pharmacogenetic testing has become more readily available for other genes relevant to drug dose individualization. Common medications prescribed by gastroenterologists for conditions other than inflammatory bowel disease now have actionable guidelines, which can improve medication efficacy and safety; however, a clear understanding of how to interpret the results remains a challenge for many clinicians, precluding wide implementation of genotype-guided dosing for drugs other than 6-mercaptopurine and azathioprine. Our goal is to provide a practical tutorial on the currently available pharmacogenetic testing options and a results interpretation for drug–gene pairs important to medications commonly used in pediatric gastroenterology. We focus on evidence-based clinical guidelines published by the Clinical Pharmacogenetics Implementation Consortium (CPIC^®^) to highlight relevant drug–gene pairs, including proton pump inhibitors and selective serotonin reuptake inhibitors and cytochrome P450 (CYP) 2C19, ondansetron and CYP2D6, 6-mercaptopurine and *TMPT* and Nudix hydrolase 15 (*NUDT15*), and budesonide and tacrolimus and CYP3A5.

## 1. Introduction

Personalized medicine and individualized drug dosing based on a person’s genetics grant many potential advantages to the patient, prescriber, and society. Customization and individualization of disease prevention and treatment allow clinicians to prescribe more effective medications while avoiding toxicity and mitigating the risk of side effects. Additionally, individualization of therapy may reduce the time and cost of trial-and-error approaches to finding the right medication at the right dose for a particular patient. Increasing interest in the utilization of pharmacogenetic information in personalized medicine helps clinicians utilize the best treatment for each patient [1,2,3]. Clinician and prescriber understanding of the basic principles of the contribution of pharmacogenetics to pharmacokinetics (drug disposition, including systemic and/or local drug concentration) and pharmacodynamics (drug effect) is integral to the incorporation of personalized medicine into mainstream, real-world, medical practice. Many pediatric gastrointestinal (GI) diseases are treated with medications that can be affected by variations in genes relevant to drug pharmacokinetics (PK) and pharmacodynamics (PD).

Both PK and PD properties of medications can contribute to their efficacy, as well as toxicity and adverse effects [4]. PK describes the drug absorption, distribution, metabolism, and elimination that drive the overall dose exposure in the body. PD is the biochemical and physiologic effects of the medication in the body (i.e., efficacy, toxicity, and adverse effects). The dose–response relationship is the interrelation between the dose of a medication administered, the PK that dose achieves for a given patient, and the subsequent overall drug response, either therapeutic or toxic. Dose–response relationships are reliant on both the PK and PD properties of a medication. Pharmacogenetics as a field investigates how an individual’s genes affect these dose–response relationships through both PK and PD mechanisms. PK/PD models can be utilized to determine the pediatric dosage requirements to optimize therapy and should be included as future models are developed. The inclusion of pharmacogenetics within these models may also improve medication dosage selection for the pediatric population. 

Clinical guidelines are available from groups such as the Clinical Pharmacogenetics Implementation Consortium (CPIC^®^) [5] and the Dutch Pharmacogenetics Working Group (DPWG) to provide suggested dosage adjustments and/or drug selection based on pharmacogenetic results. CPIC^®^ and DPWG are groups dedicated to curating peer-reviewed and evidence-based studies into easy-to-use guidelines to facilitate the dissemination and use of this information to prescribers providing direct patient care. Currently, most recommendations are based on polymorphically expressed genetic variants in the enzymes and transporters impacting the PK component of the drug dose–response relationship. Though the interaction of the drug with the drug target at the site of drug action is vital for achieving the desired therapeutic response, a paucity of evidence limits formal guidance regarding pharmacogenetic testing for PD. Thus, our focus will be on gene–drug pairs that are known to affect the pharmacogenetics (PGx) → PK/PD relationship.

### 1.1. Pharmacogenetic Test Panels

Pharmacogenetic (PGx) test panels can be utilized by clinicians to receive a succinct accounting of genetic variants of interest relevant to drug dose selection. Research is always ongoing to understand these interactions; thus, the level of evidence for each gene–drug pair varies in these reports. Understanding the differences between the level of evidence for each gene–drug pair on a test panel can help guide the value of implementing the suggested adjustment in therapy recommended. Test panel reports will detail two components for each patient, genotype and phenotype, and will include both gene and protein names. Gene names are italicized, but protein names are written in straight text. Sometimes the names of genes and the proteins they encode are identical (e.g., *CYP3A4* gene and CYP3A4 protein), but other times they differ between the gene name (e.g., *SLC01B1*) and the name of the protein that that gene encodes (e.g., OATP1B1).

PGx test panels frequently include the genotype and predicted phenotype for each gene tested. A genotype will have the name of the gene (e.g., *CYP3A4*) followed by two numbers representing the two alleles present (e.g., CYP3A4 *1/*1). For CYP enzymes, the *1 (pronounced “star one”) allele is designated as the wild type and is said to have “normal” function. This default allele is used to identify single nucleotide polymorphism (SNP) variants which are then designated by a different star number (i.e., *17). If a patient has none of the tested SNP variants, they are assigned the wild-type *1 allele designation by default. It is important to recognize that this wild-type or normal function designation may change if more SNPs are added to the panel and/or become discovered through research. For example, a patient will be designated as having two wild-type alleles if no SNPs are found; *not* testing for a specific SNP will also default to wild-type designation in the report. For some genes, there are dozens of known SNPs; thus, it is not possible to summarize all known SNPs for every gene discussed in this review. Readers are referred to https://www.pharmvar.org/genes as a resource to determine the relative functionality of each SNP in comparison to the *1 allele for each gene. Each company’s panel of tested SNPs is not identical, so care should be taken to choose a panel that is robust enough for the alleles of interest/relevance. A PGx panel’s complement of tested SNPs may be determined by various practical reasons such as cost and known frequency for SNPs within a population likely to utilize that panel. 

The genotype is strictly reporting what gene polymorphisms are present; however, the predicted phenotype is the PGx panel’s interpretation of how that genotype will affect the activity of the protein that gene encodes. In PGx panels, the reported phenotype or activity level is based on the genetic variants (i.e., SNPs) tested for, population studies of other individuals with that genotype, and as it relates to other phenotypes. Table 1 summarizes the commonly used phenotype nomenclature for drug-metabolizing enzymes. 

For each patient, other factors outside of their genotype can affect their real-world phenotype—common factors are age and the ontogeny of each individual enzyme, ethnicity, and/or interactions with other drugs or foods. GI diseases also have the unique challenge of frequently affecting the site for oral systemic medication dissolution (the stomach) and absorption (the small intestine)—adding another layer of complexity and interindividual variability to genotype-predicted phenotype. Thus, the phenotype interpretation on the panel should be considered as the patient’s starting point for dosing, rather than the sole consideration, with additional consideration of patient age, disease effect, polypharmacy, etc. Phenoconversion is the concept that at a given point in time, a person’s phenotype may not be consistent with their genotype due to age, ontogenic trajectory of the enzyme, or drug–drug/drug–food interactions, etc. For example, a one-month-old infant may have a genotype consistent with an ultra-rapid CYP2C19 metabolizer; however, based on age, their phenotype at that given age may not be equivalent to an adult ultra-rapid metabolizer. Unfortunately, there is a paucity of data on phenoconversion, especially for neonates and infants. Although sources of interindividual variability other than genetics are important to consider for precision dosing, genotype-guided drug dose selection is a good starting point and the focus of this review. 

### 1.2. Pharmacokinetic vs Pharmacodynamic Gene–Drug Pairs 

Gene–drug pairs that could necessitate drug dosing adjustments for efficacy of medications used in pediatric gastroenterology include thiopurine S-methyltransferase nucleotide diphosphatase (*TPMT*), Nudix hydrolase 15 (*NUTD15*), Cytochrome P450 enzymes (CYPs), and Uridine-diphosphate Glucuronosyltransferases (UGTs). Other genes impacting the biochemical and physiologic effects of medications include those encoding medication receptors (ex. *HTR2A, HTR2C, Grik4*) and transporters (ex. *SLC6A4*, and *SLCO1B1*); however, apart from *SLCO1B1* (discussed later), the level of evidence for actionable prescriber decision making based on these latter genes is low. 

Commonly prescribed medications for gastrointestinal complaints in children for which actionable CPIC^®^/DPWG guidelines are available include proton pump inhibitors (PPIs), selective serotonin reuptake inhibitors (SSRIs), amitriptyline, ondansetron, tacrolimus, azathioprine, and mercaptopurine (Table 2). Polyethylene Glycol 3350, lactulose, famotidine, fluconazole, ursodiol, and biologics frequently used in the treatment of inflammatory bowel disease are not extensively metabolized by the liver and are, thus, excluded from our discussion, as they do not have actionable PGx → PK → PD dates for evaluation. Medications eliminated primarily by the kidneys would not be impacted by pharmacogenetic metabolism and are, therefore, excluded. Other medications, despite metabolism via similar CYP enzymes, have no current guidelines to guide dosing, largely from lack of data.

## 2. Proton Pump Inhibitors

Proton pump inhibitors (PPIs) are acid suppressive medications that are commonly prescribed to children for treating gastroesophageal reflux disease, abdominal pain, esophagitis, and *Helicobacter pylori* infection, with regulatory approval for use in children 1 year of age or older [6]. Except for rabeprazole, the medications in this drug class (i.e., omeprazole, lansoprazole, pantoprazole, esomeprazole, and dexlansoprazole) are substrates for CYP2C19. Based on an extensive body of literature showing interindividual variability in systemic drug exposure from a given dose of a PPI [7], CPIC^®^ provides guidelines to guide appropriate drug dose selection of PPIs based on *CYP2C19* genotype. Evidence is strongest for the first generation of PPIs, which became available on the market in the late 1980s and early 1990s (i.e., omeprazole, lansoprazole, pantoprazole), with less information available for the newer, second generation of PPIs (i.e., esomeprazole, dexlansoprazole). A lack of consistent findings regarding the effect of the *CYP2C19* genotype on the PK and PD of esomeprazole has precluded CPIC from including this second-generation PPI in the current guidelines.

PPI dose adjustment recommendations based on *CYP2C19* genotype from CPIC^®^ are summarized in Table 3. The benefit of using such genotype-based dose selection is twofold. First, it enables up-front identification of patients with *CYP2C19* genotypes predictive of lower than expected plasma drug concentrations from standard dosing (i.e., individuals with copies of functional and/or supra-functional *CYP2C19* alleles; normal metabolizer/rapid metabolizer/ultra-rapid metabolizer phenotype) who are at risk for therapeutic failure without appropriate dose escalation by 50-100% to achieve clinical efficacy [7]. Second, it decreases the likelihood of PPI-associated adverse events from chronic therapy (i.e., >12 weeks) by advocating for a 50% dose reduction for individuals with one or two copies of the non-functioning allele whose genotypes are predictive of higher than expected plasma drug concentrations from standard dosing, and potential toxicity [7]. 

Table adapted from the CPIC^®^ Clinical Guidelines [7]. Classification of recommendation strength (i.e., optional vs. moderate) is based on the quality and quantity of available literature to support the recommendation at the time of guideline publication; periodic updates from CPIC^®^ are expected as new/additional publications become available. Although the CPIC^®^ guidelines recommend consideration of 50-100% dose escalation for individuals with CYP2C19 normal (NM), rapid (RM), and ultra-rapid metabolizer (UM) status only for select conditions (e.g., *H. pylori* infections, erosive esophagitis) for which ample published data were available, it is the opinion of the authors that these recommendations can and should be extended to other clinical indications for PPI use (e.g., eosinophilic esophagitis, gastroesophageal reflux disease) for which less published data was available at the time of guidelines publication. CYP2C19 metabolizer status denoted as “(likely)” encompasses novel alleles that are likely to be associated with a particular metabolizer status, but expert consensus has not yet been reached.

In the last two decades, a spectrum of unanticipated PPI-associated adverse events have come to light in both adults and children, especially with long-term PPI use [8]. For children, specifically, these include enteric [9,10] and respiratory infections [11,12], allergic [13] and sinopulmonary [14] symptoms, asthma [15], fractures [16], and most recently, anxiety and depression [17]. At least some of these adverse events (e.g., respiratory) occur more commonly in children with the *CYP2C19* intermediate or poor metabolizer phenotype [11], and are less commonly observed in children with the *CYP2C19* rapid or ultra-rapid metabolizer phenotype [12]. Thus, the risk of these adverse effects can be mitigated by genotype-guided PPI dosing [14]. In our opinion, stratification of adverse events by *CYP2C19* genotype in prior and future studies will help us better understand the emerging landscape of PPI-associated adverse events. 

Although the CPIC^®^ clinical guidelines specifically recommend dose escalation considerations for individuals with the normal, rapid, and ultra-rapid metabolizer *CYP2C19* phenotype in the setting of select clinical diagnoses (e.g., *H. pylori* infection, erosive esophagitis; Table 3), it is the opinion of these authors that these actionable recommendations apply to other conditions for which PPIs have become standard treatment (e.g., GERD, PPI-responsive eosinophilic esophagitis).

## 3. Antidepressants

Though originally used as antidepressants, selective serotonin reuptake inhibitors (SSRIs) and tricyclic antidepressants (TCAs) are now prescribed off-label for multiple, overlapping, additional indications [18]. In addition to depression, SSRIs are used with varying efficacy in the management of functional abdominal pain disorders, including irritable bowel syndrome (IBS), functional dyspepsia, and abdominal migraine [19,20]. SSRIs and TCAs can be beneficial for relieving global irritable bowel syndrome (IBS) symptoms, including decreasing abdominal pain [21]. TCAs are recommended for treating patients with diarrhea-predominant IBS (IBS-D) [22]. Multiple genes related to the PD and PK of the SSRIs and TCAs are currently available on PGx panels. 

SSRIs: SSRIs are extensively metabolized in the liver by the CYP enzymes. Each SSRI may have one or more CYP enzymes responsible for its metabolism, and each enzyme may vary in its contribution. Citalopram and escitalopram (the S enantiomer of citalopram) are metabolized by CYPs 2C19, 3A4, and 2D6 [23,24,25]. Plasma concentrations of citalopram are affected substantially by allelic variants in *CYP2C19* and the CYP2C19 metabolizer status. *CYP2C19* poor metabolizers (e.g., 2 null-function alleles like *2 and *3), for example, have reduced drug clearance [26,27,28,29,30,31] and higher systemic exposures from a given SSRI dose than “the average patient”.

Meanwhile, ultra-rapid metabolizers have increased clearance and lower systemic exposures from the same drug dose than “the average patient” [27]. Current CPIC^®^ guidelines for citalopram and escitalopram recommend a lower starting dose, and a 50% reduction in the standard maintenance dose of these drugs for *CYP2C19* poor metabolizers. Titration to a higher maintenance dose of citalopram/escitalopram or consideration of an alternative medication not predominately metabolized by *CYP2C19* could also be considered for CYP2C19 ultra-rapid metabolizers [32].

In addition to CYP2C19, the SSRI sertraline is also metabolized by multiple other enzymes, including CYPs 2B6, 2C9, 2C19, 2D6, and 3A4, as well as monoamine oxidases A and B and UGT2B7 [33,34]. Despite many enzymatic contributors, the current dosing adjustment for sertraline is guided by contributions from *CYP2C19* and *2B6* polymorphisms, as there is insufficient evidence for dosing recommendations based on other contributing pathways. For those with the *CYP2C19* ultra-rapid phenotype (e.g., *17/*17), normal dosing is suggested for initial therapy with close monitoring for lack of response, in which case an alternative agent may be required. For the CYP2C19 poor metabolizer phenotype (e.g., *2/*2), with no CYP2C19 activity, a dosage reduction is suggested [32]. The reader is referred to www.pharmGKB.org for detailed combined phenotype dosage guidance. 

The SSRI fluoxetine is metabolized by CYPs *2D6*, *2C19*, *2C9*, *3A4*, and *3A5* [35,36]. Despite *CYP2D6* being a major contributor to the metabolism of fluoxetine, it is also a potent inhibitor of this enzyme, making dosing recommendation tenuous. No gene–drug dosing recommendations are provided in the newly updated CPIC^®^ recommendation [32] due to a lack of data regarding the impact of CYP2D6 phenotype status on the sum of fluoxetine and norfluoxetine concentrations over time, related to patient outcomes or safety. In the presence of such equipoise, the drug package labeling only focuses on individuals without functional *CYP2D6* alleles (i.e., poor metabolizers), stating that dosage adjustments are not required in *CYP2D6* poor metabolizers [37].

In addition to the drug metabolizing enzymes discussed above, the genotype/phenotype of drug receptors and transporters also influence the PD of SSRIs. SSRIs bind to the solute carrier family 6, member 4 (*SLC6A4*) transporter, also known as SERT, which blocks serotonin reuptake and increases synaptic serotonin concentrations [38]. To date, the efficacy data for SSRIs and this transporter in patients with depression has shown variable outcomes [39]. SSRIs also interact with glutamate ionotropic receptor kainate type subunit 4 (*GRIK4*), 5-hydroxytryptamine receptor 1A (*HTR1A*), and 5-hydroxytryptamine receptor 2A (*HTR2A*) to contribute pharmacologic effects [40,41,42,43,44]. Evidence for these receptors is also conflicting. Though SSRIs are used off-label to treat gastrointestinal symptoms of IBS, a high prevalence of comorbid anxiety and depression in these patients makes evaluation of their efficacy particularly challenging [45]. To our knowledge, no data are available to guide PGx testing of SSRIs for gastrointestinal indications such as IBS. 

PGx data are available to suggest a relationship between transporter pharmacogenetics and patient propensity for adverse effects associated with SSRI use. Preliminary studies suggest that the “S” allele of *SLC6A4* is associated with an increased risk of insomnia and agitation, whereas the “L” allele is associated with sexual dysfunctions [46,47]. An association between the *HTR2A* rs6311 T/T genotype and the development of sexual dysfunction is seen in patients being treated for major depressive disorder [47]. The HTR2A receptor literature shows varying levels of adverse effects: some studies find patients with the rs6311 genotype C/C to suffer more nausea/vomiting, whereas others find less risk of diarrhea, dizziness, and tremor associated with the C/C genotype [48,49]. 

TCAs: Amitriptyline, a tricyclic antidepressant, is a mixed serotonin and norepinephrine reuptake inhibitor used for many conditions, including major depressive disorder, neuropathic pain, migraine prophylaxis, irritable bowel syndrome, and functional dyspepsia. Amitriptyline is a prodrug requiring demethylation to the active metabolite nortriptyline, which occurs primarily via CYP2C19, although CYPs 1A2, 2C9, 2D6, and 3A4 have all been shown to demethylate amitriptyline in vitro [50,51,52,53]. Both amitriptyline and nortriptyline are hydroxylated by CYP2D6 into less active metabolites [54,55] and excreted in the urine mainly as glucuronide conjugates with less than 5% eliminated unchanged [56]. 

The *CYP2C19* genotype has been shown to influence serum nortriptyline concentrations and PK [50,53,57,58,59], but not clinical efficacy or PD of amitriptyline [57,60]. The CYP2C19 *17 allele confers increased metabolism—homozygous patients are ultra-rapid metabolizers, whereas heterozygous patients are rapid metabolizers. This increased activity may be associated with a higher incidence of adverse events due to increased nortriptyline concentrations [61]. CYP2C19 *2 and *3 are loss of function alleles, which [62] result in increased serum amitriptyline concentrations, but little evidence links these variants alone to increased adverse effects, possibly because alternative metabolic pathways compensate for the loss of CYP2C19 function [53]. 

*CYP2D6* variations also affect the overall serum concentration of amitriptyline [50,58,59,63]. Loss of function alleles *3, *4, *5,*6 and decreased function alleles *9, *10, and *41 result in an increased risk of anticholinergic/gastrointestinal and mental adverse effects [57,64], whereas gene duplications (*1xN or *2xN allele) may lead to decreased efficacy [65,66] and the need for higher doses [66]. 

Low-dose amitriptyline is effective for functional gastrointestinal disorders such as IBS-D and functional dyspepsia [19,67]. Pharmacogenetics may not play as dominant of a role when using low-dose amitriptyline, as poor or intermediate metabolizers are less likely to experience adverse effects [58,68] and any substantial differences in clinical efficacy [60]. Thus, genotyping may be of lesser value for prescribing these drugs off-label for GI indications.

In summary, based on the evidence above, clinical guidelines recommend the use of an alternative psychiatric medication for both rapid/ultra-rapid and poor metabolizers for CYP2C19 and 2D6 [63,69,70] in place of TCAs for major depressive disorder. A 25% reduction in the TCA dose may be considered when patients are a CYP2D6 intermediate metabolizer and/or a CY2C19 normal or intermediate metabolizer. However, the above recommendations apply to depression treatment indications, and dose modifications are not currently recommended when using low-dose amitriptyline for GI indications [69]. 

## 4. Antiemetics

Ondansetron is a selective 5-HT_3_ receptor antagonist used to prevent postoperative, chemotherapy-induced, and radiotherapy-associated nausea and vomiting; however, it is also used off-label for nausea/vomiting in general. It is metabolized into four inactive metabolites, primarily by CYP2D6, with CYPs 3A4 and 1A2 contributing to a lesser extent [71,72].

Pharmacogenetic studies have primarily focused on variations in *CYP2D6* and their impact on ondansetron PK and PD. Patients with three functional copies due to gene duplication of the *CYP2D6* allele metabolize ondansetron more quickly, leading to decreased efficacy, increased nausea, and vomiting [73,74,75,76,77]. Selecting an alternative agent not primarily metabolized by *CYP2D6*, such as granisetron, is recommended [78].

Ondansetron produces a similar clinical response in CYP2D6 poor metabolizers as in normal and intermediate metabolizers [73,74,75]; however, serum concentrations are higher in those with *CYP2D6* loss of function and decreased function alleles compared to normal metabolizers [77]. Data are lacking on the association of increased serum concentration and increased adverse effects, so dosage reduction is not currently recommended for poor or intermediate metabolizers at this time [78]. 

## 5. Immunosuppressants

Tacrolimus and cyclosporine are immunosuppressants utilized to prevent and treat allograft rejection in intestinal and liver transplants. Both medications are calcineurin inhibitors, although tacrolimus is significantly more potent [79]. Both drugs are extensively metabolized by CYP3A4 and CYP3A5 in the liver, as well as in the epithelium lining the GI tract [80,81,82,83]. For tacrolimus, CYP3A5 is the predominant enzyme for metabolism, with lower rates of metabolism via CYP3A4 [84,85], whereas cyclosporine is primarily metabolized by CYP3A4 [86]. 

In addition to CYP3A4 and CYP3A5, the efflux transporter P-glycoprotein (P-gp, *ABCB1*) also plays a major role in the PK of tacrolimus and cyclosporine [87,88]. However, the majority of pharmacogenetic studies on tacrolimus and cyclosporine have focused on the effects of genetic variants of *CYP3A4*, *CYP3A5* and *ABCB1,* and currently, there are no dose adjustments recommended specifically for the P-gp phenotype. Although this transporter is crucial to the PK of these medications, studies thus far have not identified genetic variants that consistently contribute to PK or clinical outcomes (PD). Therefore, we will focus our discussion on *CYP3A4* and *CYP3A5*.

Unlike other CYP enzymes, where the wild type is the most common genotype, the most common genotype for *CYP3A5* in the United States is *3*3, with two copies of the loss of function allele (i.e., CYP3A5 poor metabolizer phenotype). This poor metabolizer (PM) phenotype is most prevalent in the Caucasian population, with intermediate (*1*3) or normal metabolizer (*1*1) phenotypes significantly more common in patients with African or Asian ancestry [89]. Current CPIC^®^ recommendations [90] for those with a *1*3 or *1*1 CYP3A5 genotype are to consider a tacrolimus starting dosage increase of 1.5–2 times of the recommended starting dose, but not exceeding a total starting dose of 0.3 mg/kg/day. Therapeutic drug monitoring should be utilized to make dose adjustments. Since the CYP3A5*6 and *7 alleles are rare, the impact of these alleles on the trough levels of tacrolimus have only been studied in combination with the CYP3A5*3 allele. 

For patients undergoing liver transplant, the genotype of the *donor,* rather than the recipient, will guide the majority of tacrolimus and cyclosporin drug metabolism in the liver and, therefore, dosing decisions should be made based on the donor CYP3A genotype. PGx testing of the donor could conceivably assist with tacrolimus dosing upon initiation, though dosage adjustment based on serum drug concentrations should still be utilized. Intuitively, the genotype of the donor, not the recipient, could also influence the PGx–PK–PD relationship for other drugs and drug metabolizing enzymes; however, data are lacking for the majority of gene–drug pairs.

Azathioprine and mercaptopurine are immunosuppressant thiopurine antimetabolites used for nonmalignant immunologic disorders such as inflammatory bowel disease and autoimmune hepatitis [90]. Azathioprine is a prodrug of mercaptopurine, and both drugs are metabolized by multiple enzymatic pathways, including thiopurine S-methyltransferase (*TPMT*) and nucleotide diphosphatase (Nudix hydrolase 15, *NUDT15*) [90,91,92,93,94,95,96,97,98,99,100,101,102,103]. Allelic variants in *TPMT* and *NUDT15* can shift azathioprine/mercaptopurine metabolism toward higher production of the active metabolites thioguanine-nucleotides (6-TGNs). Although adequate 6-TGN levels are needed to achieve a therapeutic effect, an overabundance of 6-TGNs can lead to over-suppression of the immune system, resulting in the unwanted and potentially serious side effect of bone marrow suppression. Therefore, dose reduction is recommended for individuals with allelic variants encoding decreased *TPMT* and *NUDT15* function, to avoid bone marrow toxicity. The CPIC^®^ recommendations for non-malignant conditions are that alternative therapy may need to be considered for individuals homozygous for *TPMT*/*NUDT15* loss of function alleles [90]. In addition to genotype, phenotype testing is available to measure *TPMT* enzymatic activity in red blood cells. This testing directly reports phenotype activity rather than inferring it based on genotype. Interindividual variations in *TPMT* activity are low, so repeated testing is not required [94]. 

Society guidelines and the U.S. Food and Drug Administration (FDA) [92,95,96] recommend *TPMT* testing prior to initiation of thiopurine therapy, preferably with phenotypic testing [97]. Though genotypic testing will not be affected by recent blood transfusions or drug/food interactions, some patients with bone marrow suppression are not found to have low functioning alleles [98]. Thus, for the practicing clinician, phenotypic testing is often more relevant and actionable, compared to genotype testing. Additionally, the American College of Gastroenterology suggests *TPMT* genotyping or phenotyping for thiopurine dose guidance, and therapeutic drug monitoring of metabolites 6-MMP and 6-TGN for subsequent dosage adjustments. Although the guidelines specifically call out therapeutic drug monitoring (TDM) for those individuals who are unresponsive or refractory to treatment—presumably due to inadequate systemic 6-TG despite standard dosing—and those with adverse effects, such as hepatotoxicity related to an overabundance of 6-MMP, it is our opinion that TDM, if available, should be used for all patients. Of note, genotype/phenotype testing and TDM should be used in conjunction with, not in place of, routine monitoring of blood counts and liver enzymes in patients receiving thiopurines [92,95]. 

## 6. Corticosteroids

Budesonide is a corticosteroid used in oral and inhaled preparations, and valued for its first-pass metabolism in the liver, which decreases side effects from systemic corticosteroid exposure (growth failure, iatrogenic adrenal insufficiency) commonly seen with other steroids [99]. Budesonide is used as a locally active, topical anti-inflammatory for inflammatory bowel disease and eosinophilic esophagitis [100]. Similar to the aforementioned calcineurin inhibitors, budesonide is metabolized through CYP3A4 and CYP3A5 in the liver and gastrointestinal tract into inactive metabolites 6b-hydroxybudesonide and 16a-hydroxyprednisolone [102]. Additionally, budesonide is also a substrate for transporter P-gp. 

As mentioned above, CYP3A4 and CYP3A5 enzymes can be found in the intestinal epithelium, where their expression is influenced by inflammation [103] in addition to genotype. Since budesonide is used to treat local inflammation, local (rather than hepatic) drug metabolism modulated by inflammation at that location may contribute more significantly to medication efficacy than first-pass metabolism in the liver. GI inflammation, through its downregulation of CYP3A expression [103] and function [104] locally, may contribute to higher systemic exposure of orally administered budesonide [105,106]. Small, preliminary studies also indicate that the *CYP3A5* genotype may contribute to budesonide treatment failure in eosinophilic esophagitis for patients with wild-type alleles who may metabolize the drug too quickly [107]. Further studies evaluating the contribution of the *CYP3A5* genotype, *ABCB1* genotype, and esophageal inflammation on the treatment of GI diseases with budesonide are under way.

## 7. Conclusions

In summary, pharmacogenetic testing can help avoid trial-and-error treatment approaches for patients and minimize the potential for medication side effects and toxicities, while optimizing timely therapeutic response. Pharmacogenetics can also identify patients who are unlikely to gain any therapeutic benefit from a specific drug agent, expediting their treatment path by starting with a more appropriate alternative and avoiding time-consuming trial-and-error approaches. Current evidence indicates that pharmacogenetic testing can aide with the initial drug dose selection of PPIs except rabeprazole (*CYP2C19* testing), some SSRIs (*CYP2C19* and *CYP2D6*), tricyclic antidepressants (*CYP2C19* and *CYP2D6*), and the calcineurin inhibitors tacrolimus and cyclosporin (*CYP3A4/5*). Recommendations for testing for corticosteroids (*CYP3A5*) are still evolving. Pharmacogenetic testing can also identify those patients who are likely to experience adverse events from ondansetron (*CYP2D6* ultra-rapid metabolizers with three functional copies of *CYP2C6*) and who would therefore benefit from a selection of an alternative anti-emetic agent. Phenotype testing, rather than genotype testing, is more appropriate for azathioprine and 6-mercaptopurine. However, genotype information can still be helpful, particularly if phenotype testing cannot be performed (e.g., it is not covered by a third-party payer). 

As evidenced here, although some drugs offer a wealth of information for guidance, others suffer from a paucity of reliable studies. Additionally, real-world medical practice brings new complications that may affect the ultimate phenotype of patients. Inflammation from disease at the site of drug uptake will affect receptor, transporter, and enzyme presence and efficiency. Even at a baseline healthy status, the GI tract is a milieu of ever-changing food, bacteria, and secretions interactions with orally administered drugs that can influence PK and PD, in addition to PGx. Pharmacogenetics provides great guidance, but like all aspects of precision therapeutics, each individual patient’s clinical scenario should be carefully considered to make rational individually targeted therapeutic decisions. 

## Figures and Tables

**Table 1:** Standard phenotype nomenclature.

Phenotype	Allele Activity
Ultra-Rapid Metabolizer (UM)	Two supra-functional alleles
Rapid Metabolizer (RM)	One normal function and one supra-functional allele
Normal Metabolizer (NM)	Two normal function alleles (i.e., wild type)
Intermediate Metabolizer (IM)	One loss/null function allele and one normal/supra-function allele
Poor Metabolizer (PM)	Two loss/null function alleles

**Table 2:** Medications commonly prescribed for pediatric gastrointestinal complaints with actionable CPIC^®^/and/or DPWG guidelines.

Actionable CPIC^®^ and/or Dutch Working Group Guidelines Available	Not Actionable Lack Significant HEPATIC Metabolism/Predominant Renal Elimination	More Research Needed No Current Guidelines or Recommendations
* Proton Pump Inhibitors (CYP2C19) * Selective Serotonin Reuptake Inhibitors (CYP2C19, CYP2D6, CYP2B) * Amitriptyline (CYP2C19, CYP2D6) * Ondansetron (CYP2D6) * Tacrolimus (CYP3A5) * Azathioprine (TPMT, NUDT15) * Mercaptopurine (TPMT, NUDT15)	Miralax Lactulose Famotidine Fluconazole Ursodiol Biologics	Budesonide (CYP3A4, CYP3A5) Fluticasone (CYP3A4) Erythromycin (CYP3A4) Lorazepam (UGT2B15) Diphenhydramine (CYP2D6) Sirolimus (CYP3A4, CYP3A5) Micafungin (CYP3A4, COMT) Cimetidine (CYP1A2, CYP2C19) Cyproheptadine (multiple UGTs) Prednisone (CYP3A4) Mycophenolate mofetil (multiple UGTs, SLCO1B1)

* Denotes where oral liquid formulations or extemporaneous preparations of medications with actionable guidelines may be available for easier dosage adjustments based on weight and pharmacogenetic information. For medication classes—select medications may have oral liquid formulations/extemporaneous preparations.

**Table 3:** Summary of proton pump inhibitor (PPI) dose selection recommendations from the Clinical Pharmacogenetics Implementation Consortium (CPIC^®^).

CYP2C19 Phenotype	Implications for Phenotypic Measures	Therapeutic Recommendations	Classification of Recommendation-Omeprazole, Lansoprazole, Pantoprazole	Classification of Recommendation-Dexlansoprazole
CYP2C19 UM	Decreased plasma concentrations of PPIs compared to CYP2C19 NMs; increased risk of therapeutic failure	Increase starting daily dose by 100%. Daily dose may be given in divided doses. Monitor for efficacy.	Optional	Optional
CYP2C19 RM	Decreased plasma concentrations of PPIs compared to CYP2C19 NMs; increased risk of therapeutic failure	Consider increasing dose by 50–100% for the treatment of *H. pylori* infection and erosive esophagitis. Daily dose may be given in divided doses. Monitor for efficacy.	Moderate	Optional
CYP2C19 NM	Normal PPI metabolism; may be at increased risk of therapeutic failure compared to CYP2C19 IMs and PMs	Initiate standard starting daily dose. Consider increasing dose by 50-100% for the treatment of *H. pylori* infection and erosive esophagitis. Daily dose may be given in divided doses. Monitor for efficacy.	Moderate	Optional
CYP2C19 (likely) IM	Increased plasma concentration of PPI compared to CYP2C19 NMs; increased chance of efficacy and potentially toxicity	Initiate standard starting daily dose. For chronic therapy (>12 weeks) and efficacy achieved consider 50% reduction in daily dose and monitor for continued efficacy.	Optional	Optional
CYP2C19 (likely) PM	Likely increased plasma concentration of PPI compared to CYP2C19 NMs; likely increased chance of efficacy and potentially toxicity	Initiate standard starting daily dose. For chronic therapy (>12 weeks) and efficacy achieved, consider 50% reduction in daily dose and monitor for continued efficacy.	Moderate	Optional

## Data Availability

Not applicable.
